# Brain renin-angiotensin system and dopaminergic cell vulnerability

**DOI:** 10.3389/fnana.2014.00067

**Published:** 2014-07-08

**Authors:** Jose L. Labandeira-García, Pablo Garrido-Gil, Jannette Rodriguez-Pallares, Rita Valenzuela, Ana Borrajo, Ana I. Rodríguez-Perez

**Affiliations:** ^1^Laboratory of Neuroanatomy and Experimental Neurology, Department of Morphological Sciences, CIMUS, Faculty of Medicine, University of Santiago de CompostelaSantiago de Compostela, Spain; ^2^Networking Research Center on Neurodegenerative Diseases (CIBERNED)Madrid, Spain

**Keywords:** aging, angiotensin, dopamine, NADPH-oxidase, neurodegeneration, neuroinflammation, oxidative stress, parkinson

## Abstract

Although the renin-angiotensin system (RAS) was classically considered as a circulating system that regulates blood pressure, many tissues are now known to have a local RAS. Angiotensin, via type 1 receptors, is a major activator of the NADPH-oxidase complex, which mediates several key events in oxidative stress (OS) and inflammatory processes involved in the pathogenesis of major aging-related diseases. Several studies have demonstrated the presence of RAS components in the basal ganglia, and particularly in the nigrostriatal system. In the nigrostriatal system, RAS hyperactivation, *via* NADPH-oxidase complex activation, exacerbates OS and the microglial inflammatory response and contributes to progression of dopaminergic degeneration, which is inhibited by angiotensin receptor blockers and angiotensin converting enzyme (ACE) inhibitors. Several factors may induce an increase in RAS activity in the dopaminergic system. A decrease in dopaminergic activity induces compensatory upregulation of local RAS function in both dopaminergic neurons and glia. In addition to its role as an essential neurotransmitter, dopamine may also modulate microglial inflammatory responses and neuronal OS *via* RAS. Important counterregulatory interactions between angiotensin and dopamine have also been observed in several peripheral tissues. Neurotoxins and proinflammatory factors may also act on astrocytes to induce an increase in RAS activity, either independently of or before the loss of dopamine. Consistent with a major role of RAS in dopaminergic vulnerability, increased RAS activity has been observed in the nigra of animal models of aging, menopause and chronic cerebral hypoperfusion, which also showed higher dopaminergic vulnerability. Manipulation of the brain RAS may constitute an effective neuroprotective strategy against dopaminergic vulnerability and progression of Parkinson’s disease.

## Introduction

The renin-angiotensin system (RAS) was initially considered as a circulating humoral system, with functions in regulating blood pressure and in sodium and water homeostasis. The RAS is phylogenetically one of the oldest hormone systems. It has been suggested that the RAS played an important role in human evolution, and it is possible that our ancestors may have survived on little salt, thanks to RAS activation (Lev-Ran and Porta, 2005). Angiotensin II (AII), which is the most important effector peptide of the RAS, is formed by the sequential action of two enzymes -renin and angiotensin converting enzyme (ACE)- on the precursor glycoprotein angiotensinogen. The actions of AII are mediated by two main cell receptors: AII type 1 and 2 (AT1 and AT2) receptors (Unger et al., 1996; Oro et al., 2007; Jones et al., 2008). In addition to the afore mentioned components of the RAS, several other components that are involved in secondary mechanisms of this system have emerged (Cuadra et al., 2010; Wright and Harding, 2013). The AT1 receptor mediates most of the classical peripheral actions of AII. It is generally considered that AT2 receptors exert actions directly opposed to those mediated by AT1 receptors thus antagonizing many of the effects of the latter (Chabrashvili et al., 2003; Jones et al., 2008). However, the relationships between AT1 and AT2 are probably more complex and remain to be fully clarified.

## The local (tissue or paracrine) RAS. Role in oxidative stress, inflammation and tissue degeneration

It is now known that, in addition to the “classical” humoral RAS, many tissues have local (tissue or paracrine) RAS that contain the different components previously described for the circulating RAS (Ganong, 1994; Re, 2004). Although both circulating RAS and local RAS act together in different tissues, it is generally accepted that circulating components are far less important than local formation of angiotensins for functioning of the system. Abnormal upregulation of local AII induces oxidative stress (OS) damage and exacerbates of inflammation. AII is a major activator of the NADPH-oxidase complex (Zalba et al., 2001; Touyz, 2004; Hoogwerf, 2010) which is the most important intracellular source of reactive oxygen species (ROS) other than mitochondria (Babior, 1999, 2004; Cai, 2005). It is known that NADPH-dependent oxidases are upregulated in major aging-related diseases such as hypertension, diabetes and atherosclerosis (Griendling et al., 2000; Münzel and Keany, 2001). It is usually considered that activation of AT2 receptors inhibits NADPH-oxidase activation and counteracts the deleterious effects of AT1 activation. In peripheral tissues, the upregulated AII acts, via AT1 receptors, on the resident cells (i.e., endothelial cells, smooth muscle cells) leading to OS, and subsequent production of chemokines, cytokines, and adhesion molecules, which contribute to the migration of inflammatory cells into the injured tissue (Ruiz-Ortega et al., 2001; Suzuki et al., 2003). Furthermore, AII acts on inflammatory cells to induce inflammatory responses and to release high levels of ROS mainly by activation of the NADPH complex (Okamura et al., 1999; Yanagitani et al., 1999; Qin et al., 2004; Touyz, 2004).

Finally, in addition to the “classical” humoral RAS and the local or tissue RAS, a number of recent studies support the existence of third level of RAS in several types of cells (Baker et al., 2004): the intracellular or intracrine RAS. The existence of functional intracellular RAS opens up new perspectives for understanding the effects of the RAS and for the management of RAS-related diseases (Kumar et al., 2007, 2009).

## The brain RAS. Local RAS in the nigrostriatal dopaminergic system

The role of the RAS on brain function was initially associated with effects of the circulating RAS in areas involved in the central control of blood pressure and sodium and water homeostasis, which are located in circumventricular organs lacking the blood-brain barrier (von Bohlen und Halbach and Albrecht, 2006; Phillips and de Oliveira, 2008). However, over the last two decades, all components of the classical RAS have been identified in different brain areas inside the blood-brain barrier, and the brain RAS has been suggested to be involved in additional functions and disorders (Kerr et al., 2005; Maul et al., 2005; Saavedra, 2005; Saab et al., 2007). Interestingly, it has been observed that brain levels of AII are much higher than circulating levels (Hermann et al., 1984), and that the precursor protein angiotensinogen is mainly produced by astrocytes (Stornetta et al., 1988; Milsted et al., 1990), although it is also produced at low levels in neurons (Kumar et al., 1988; Thomas et al., 1992). Major components involved in the effects of AII in peripheral tissues such as NADPH-oxidases have also been located in neurons (Noh and Koh, 2000; Wang et al., 2004) and glial cells (Gao et al., 2003; Wu et al., 2003). Several studies have shown that, as previously observed in peripheral organs, AT1 receptor blockers and ACE inhibitors (ACEIs) decreased the inflammatory response in the CNS (Platten et al., 2009; Stegbauer et al., 2009; Saavedra, 2012). In accordance with their inhibitory effect on brain inflammation, beneficial effects of AT1 inhibition have been observed in a number of processes mediated by microglial activation and neuroinflammation, including animal models of Alzheimer’s disease (Kehoe and Wilcock, 2007; Mogi and Horiuchi, 2009), brain ischemia (Lou et al., 2004; Iwanami et al., 2010) and multiple sclerosis (Platten et al., 2009; Stegbauer et al., 2009).

Several studies have reported the presence of RAS components in the basal ganglia, particularly in the nigrostriatal system (Quinlan and Phillips, 1981; Simonnet et al., 1981; Brownfield et al., 1982; Chai et al., 1987; Allen et al., 1992). In recent studies (Rodriguez-Pallares et al., 2008; Joglar et al., 2009; Valenzuela et al., 2010; Garrido-Gil et al., 2013b), we used laser confocal microscopy and other methods to demonstrate the presence of AT1 and AT2 receptors in nigral dopaminergic neurons and glial cells (i.e., astrocytes and microglia) in rodents and primates, including humans (Garrido-Gil et al., 2013b), as well as in primary mesencephalic cell cultures (Rodriguez-Pallares et al., 2004, 2008; Joglar et al., 2009). Furthermore, we demonstrated the presence of different cytoplasmatic and membrane subunits of the NADPH complex in mesencephalic dopaminergic neurons, astrocytes and microglia (Rodriguez-Pallares et al., 2007, 2008; Joglar et al., 2009). Recently, we have described, for the first time, prorenin receptors in nigral dopaminergic neurons and microglial cells in humans, monkeys and rats (Valenzuela et al., 2010; Garrido-Gil et al., 2013b). Interestingly, the labeling for prorenin, AT1 and AT2 receptors was not only located at the cell surface but also intracellularly in dopaminergic neurons and glial cells (Garrido-Gil et al., 2013b). Therefore, our observations support the existence of an intracellular/intracrine RAS in neurons, and particularly in dopaminergic neurons, as previously suggested for other cell types (Baker et al., 2004; Kumar et al., 2007, 2009).

## Increased local RAS activity enhances dopaminergic cell vulnerability: mechanisms involved

In several recent studies, we used 6-OHDA and MPTP models of parkinsonism to study the possible role of the brain RAS in dopaminergic degeneration: the results suggest that enhanced levels of AII, via AT1 receptors, exacerbate dopaminergic cell death and may play a synergistic role in the pathogenesis and progression of PD. Recent experimental data from other laboratories also support the involvement of brain RAS in dopaminergic degeneration (Grammatopoulos et al., 2007; Zawada et al., 2011; Sonsalla et al., 2013). It was observed that AII increased the neurotoxic effect induced by low doses of dopaminergic neurotoxins, and that treatment with ACEIs (Lopez-Real et al., 2005; Muñoz et al., 2006; Sonsalla et al., 2013) or blockage of AT1 receptors (Rey et al., 2007; Rodriguez-Pallares et al., 2008; Joglar et al., 2009) led to significant reduction in the loss of dopaminergic neurons and levels of protein oxidation and lipid peroxidation induced by the neurotoxins (Sanchez-Iglesias et al., 2007). Interestingly, the neuronal loss was also reduced by inhibitors of NADPH-oxidase activation, which suggests that NADPH activation and NADPH-derived ROS are involved in the AII-enhanced dopaminergic neuron death (Rey et al., 2007; Rodriguez-Pallares et al., 2008; Joglar et al., 2009).

**Figure 1:**
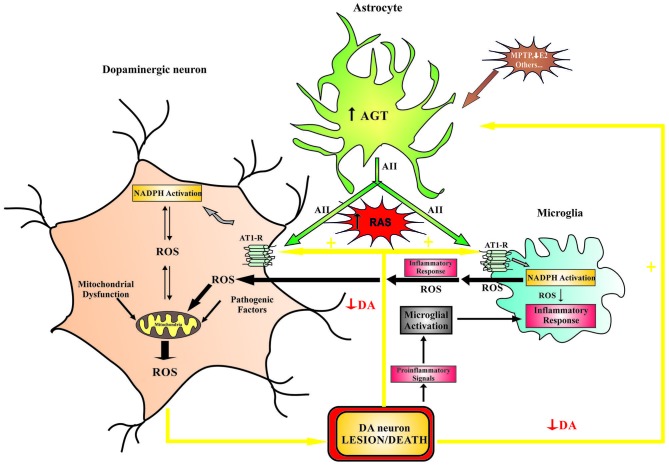
**Model of the role that brain RAS plays in dopaminergic cell vulnerability**. Different pathogenic factors (e.g., mitochondrial dysfunction, aging-related changes, neurotoxins, etc.) may initiate dopaminergic lesions and diminish dopaminergic function, which leads to increased RAS activation and progression of the dopaminergic degeneration. Furthermore, neurotoxins and proinflammatory factors may act directly on astrocytes and induce an increase in ANG/AII production, which leads to an increase in RAS activity and dopaminergic vulnerability. In dopaminergic neurons, increased RAS activity (via AT1 receptors) increases NADPH-oxidase activity, which enhances intraneuronal ROS production (in an interaction with mitochondria) and pro-inflammatory signals. In microglial cells, increased RAS activity stimulates the NADPH-oxidase complex, which enhances the inflammatory response, promoting extracellular release of high levels of ROS, activation of ROCK, and the release of cytokines and different neurotoxic factors. ANG, angiotensinogen; AII, angiotensin II; AT1, angiotensin type I receptors; DA, dopamine; E2, estrogen; NADPH, NADPH-oxidase complex; RAS, renin-angiotensin system; ROCK, Rho-associated kinase; ROS, reactive oxygen species.

In peripheral tissues (see above) and brain (Benicky et al., 2009; Wright and Harding, 2013), abnormal upregulation of local AII induces OS damage and exacerbates inflammation. Oxidative stress and neuroinflammation (including microglial NADPH-oxidase activation) constitute early components of dopaminergic cell death and both factors act synergistically with others to induce progression of PD (Gao et al., 2003; Wu et al., 2003; Rodriguez-Pallares et al., 2007). In the substantia nigra, AII receptors and NADPH-oxidase were observed in dopaminergic neurons and glial cells. Therefore, AII may also enhance dopaminergic degeneration through several mechanisms, as previously observed in the vessel wall (Figure 1). First, AII acts on neurons (i.e., resident cells) via AT1 receptors and stimulates production of low levels of intraneuronal ROS by activation of neuronal NADPH-oxidase. ROS act as second messengers in several signaling pathways, including those involved in triggering the inflammatory response and the migration of inflammatory cells into the lesioned area; NADPH-derived ROS also modulate neuronal levels of ROS by interacting with mitochondria-derived ROS and with ROS from other sources such as dopaminergic neurotoxins or activated microglia. Feed-forward cross-talk signaling between NADPH oxidase-derived ROS and mitochondria-derived ROS has been observed in several types of cells (Doughan et al., 2008; Wosniak et al., 2009). This interaction has recently been confirmed in a dopaminergic cell line treated with the neurotoxin MPP^+^ and angiotensin (Zawada et al., 2011) and in our recent studies with primary cultures of dopaminergic cells (Rodriguez-Pallares et al., 2009, 2012). Second, AII acts on microglia (i.e., inflammatory cells), in which NADPH oxidase activation produces high concentrations of ROS, which are released extracellularly and affect neurons; AII also produces low levels of microglial intracellular ROS, which act as a second messenger in several microglial signaling pathways involved in the inflammatory response (Babior, 2004; Qin et al., 2004). We have recently shown that activation of the microglial RhoA/ROCK pathway (Villar-Cheda et al., 2012a; Borrajo et al., 2014b), release of microglial TNF-α (Borrajo et al., 2014a), and altered iron homeostasis (Garrido-Gil et al., 2013a) are involved in the enhancing effect of AII/AT1 activation on the microglial response and dopaminergic degeneration. Activation of peroxisome proliferator-activated receptor gamma (PPAR-γ) also mediates the neuroprotective and anti-inflammatory effects of AT1 receptor inhibition (Garrido-Gil et al., 2012).

## Factors that may increase RAS activity in the nigrostriatal dopaminergic system

Interaction between dopamine and angiotensin was initially suggested to occur in the basal ganglia because acute administration of AII directly into the striatum (via microdialysis probes) induced an increase in extracellular levels of dopamine in normal rats, which was blocked by co-administration of AT1 receptor antagonists (Mendelsohn et al., 1993; Brown et al., 1996). This suggested that AII, via AT1 receptors, facilitates the release of dopamine. However, acute or chronic administration of AT1 receptor antagonists alone did not alter striatal dopamine levels (Dwoskin et al., 1992; Mendelsohn et al., 1993; Brown et al., 1996; Jenkins, 2008). This was attributed to possible compensatory mechanisms, which we have recently investigated in normal rats and dopaminergic denervated rats (Dominguez-Meijide et al., 2014). Therefore, it seems reasonable to assume that a decrease in dopaminergic activity may induce a compensatory increase in RAS activity to increase striatal dopamine. However, if the dopaminergic system is impaired (e.g., in the initial stages of dopaminergic lesions or aging), normal dopaminergic levels cannot be reached and the resulting overactivation of the RAS may exacerbate the microglial inflammatory response and produce OS, leading to progression of dopaminergic vulnerability and neurodegeneration. In a series of recent studies, we have confirmed that a decrease in dopaminergic activity induces compensatory upregulation of local RAS function in both dopaminergic neurons and glia (Figure 1). It is known that both angiotensin (Rodriguez-Pallares et al., 2004, 2008; Joglar et al., 2009; Garrido-Gil et al., 2013b) and dopamine (Miyazaki et al., 2004; Färber et al., 2005) receptors are located in neurons, microglia and astrocytes. In the nigrostriatal system, we observed that dopamine depletion induced a significant increase in AT1 and AT2 receptor expression, and NADPH-oxidase complex activity, which decreased as dopamine function was restored (Villar-Cheda et al., 2010). More recently, we investigated the possible interactions between angiotensin and dopamine receptors in D1-, D2-, and AT1-deficient mice, as well as mice over-expressing D2 receptors. A counter-regulatory mechanism between dopamine and angiotensin receptors was observed in the striatum and substantia nigra of these mice (Villar-Cheda et al., 2014). A similar interaction between dopamine and angiotensin receptors has recently been demonstrated in peripheral tissues, particularly in relation to the regulation of renal sodium excretion and cardiovascular function (Zeng et al., 2006; Khan et al., 2008; Gildea, 2009; Padia et al., 2012).

Other factors may induce an increase in RAS activity independently or before the loss of dopamine (Figure 1). It is known that dopaminergic neurotoxins such as MPP^+^ can act directly on astrocytes to induce an increase in production of proinflammatory factors (Henze et al., 2005; Block et al., 2007); astrocytes are the main source of angiotensinogen/angiotensin (Stornetta et al., 1988; Milsted et al., 1990), which may then act on neurons and microglial cells as indicated above. A loss of estrogen and other mechanisms that inhibit the neuroinflammatory response may also induce RAS activation and lead to an increase in dopaminergic neuron vulnerability as detailed below.

## Dopaminergic vulnerability in aging, menopause and brain hypoperfusion. Role of RAS hyperactivity

In recent studies, we investigated whether enhanced RAS activity in the nigra may be involved in the increased vulnerability of dopaminergic neurons to degeneration observed in aging, post-menopause or chronic cerebral hypoperfusion. Several studies have shown that normal aging is associated with a proinflammatory and pro-oxidant state that may favor an exaggerated response to injury and degenerative diseases (Csiszar et al., 2003; Ungvari et al., 2004; Choi et al., 2010). We have confirmed that, in aged male rats, aging enhances levels of neuroinflammation, OS markers and dopaminergic cell death induced by dopaminergic neurotoxins. The nigral RAS is involved in these effects, and levels of neuroinflammation, OS markers and dopaminergic cell death are reduced by treatment with the AT1 antagonist candesartan (Villar-Cheda et al., 2012b, 2014). Numerous studies in animal models and humans have shown aging-related loss of striatal D2 and D1 receptors (Wang et al., 1998; Ishibashi et al., 2009; Rieckmann et al., 2011), and that the dopaminergic system is altered during normal aging (Kubis et al., 2000; Collier et al., 2007). Therefore, the RAS upregulation that we observed in aged rats may be part of the compensatory changes related to decreased levels of dopamine or dopamine receptors (Villar-Cheda et al., 2012b, 2014). However, other factors may also be involved (Cruz-Muros et al., 2007, 2009), as aging has been shown to be associated with overactivation of RAS in a number of tissues (Thompson et al., 2000; Min et al., 2009; Cassis et al., 2010). Thus, the upregulation of AT1 receptors observed in aged rats may be part of the compensatory changes related to changes in the dopaminergic system; however, the compensatory upregulation of AT2 receptors observed in young rats with similar changes in the dopaminergic system was not observed in aged rats.

Menopause has also been identified as a prominent risk factor for PD. Numerous experimental studies have shown that estrogen exerts protective effects against dopaminergic cell degeneration (Leranth et al., 2000; Callier et al., 2002). The anti-inflammatory effects of estrogen play a major role in the neuroprotective effects (Suzuki et al., 2007; Vegeto et al., 2008), although direct anti-apoptotic (Das et al., 2011; Brendel et al., 2013) and trophic (López-Martín et al., 1999; Campos et al., 2012) effects on neurons have also been suggested. A number of epidemiological studies have also reported that the incidence and prevalence of PD is higher in postmenopausal women and men than in premenopausal women of similar age (Currie et al., 2004; Ragonese et al., 2006a,b). However, some reported effects of estrogen replacement therapy are controversial (Shulman, 2002; Popat et al., 2005), and the age and duration of lack of estrogen in women receiving the treatment appear to be major factors in the discrepancies. Interestingly, estrogen-induced regulation of the RAS mediates beneficial effects of estrogen in several tissues (Nickenig et al., 1998; Dean et al., 2005; Chen et al., 2008), and interactions between estrogen and AII receptors have also been observed (Liu et al., 2002; Tsuda et al., 2005; Xue et al., 2007; Hoshi-Fukushima et al., 2008). In several recent studies we have observed that the lack of estrogen increases RAS activity in the substantia nigra in females (Rodriguez-Perez et al., 2010, 2011, 2012). We compared young ovariectomized rats (i.e., early surgical menopause) with aged rats (i.e., natural menopause). Both groups of menopausal rats showed increased RAS activity. However, estrogen therapy significantly reduced 6-OHDA-induced dopaminergic cell loss in young rats but not in aged rats, suggesting that other factors are involved in aged females. Interestingly, treatment with the AT1 antagonist candesartan significantly reduced dopaminergic neuron loss in both groups of menopausal rats (Rodriguez-Perez et al., 2012).

Dopaminergic cell loss and signs parkinsonism have been observed in elderly people without PD (almost 40%; Buchman et al., 2012), and presynaptic dopaminergic function is reduced in most patients with vascular parkinsonism (Zijlmans et al., 2007), suggesting an interaction between aging-related cerebrovascular disease/brain hypoperfusion and dopaminergic degeneration. This was confirmed in a recent study with animal models of chronic brain hypoperfusion (Rodriguez-Perez et al., 2013), in which we have shown that chronic hypoperfusion increases dopaminergic cell death by enhancing the deleterious effects of other factors (such as low doses of dopaminergic neurotoxins). This suggests that hypoperfusion derived from aging and/or vascular disease may increase the risk of development of parkinsonism. The mechanistic links between hypoperfusion/vascular disease and neurodegeneration are unknown. However, chronic hypoperfusion led to increased expression of inflammatory markers such as IL-1β and increased levels of OS markers such as NADPH activity (Rodriguez-Perez et al., 2013), which have been shown to be involved in the progression of dopaminergic cell death in animal models of PD and in PD patients (Wu et al., 2003; Koprich et al., 2008). Interestingly, these changes were accompanied by increased RAS activity in the substantia nigra, and they were inhibited by chronic treatment with the AT1 receptor antagonist candesartan (Rodriguez-Perez et al., 2013).

## Conflict of interest statement

The authors declare that the research was conducted in the absence of any commercial or financial relationships that could be construed as a potential conflict of interest.
